# Super-hydration and reduction of manganese oxide minerals at shallow terrestrial depths

**DOI:** 10.1038/s41467-022-29328-y

**Published:** 2022-04-11

**Authors:** Seohee Yun, Huijeong Hwang, Gilchan Hwang, Yeongkyoo Kim, Douglas Blom, Thomas Vogt, Jeffrey E. Post, Tae-Yeol Jeon, Tae Joo Shin, Dong-Zhou Zhang, Hiroyuki Kagi, Yongjae Lee

**Affiliations:** 1 Department of Earth System Sciences, Yonsei University, Seoul, 03722 Republic of Korea; 2 School of Earth System Sciences, Kyungpook National University, Daegu, 41566 Republic of Korea; 3 Nano Center and Department of Chemical Engineering, University of South Carolina, Columbia, SC 29208 USA; 4 Nano Center, Departments of Chemistry & Biochemistry and Chemical Engineering, University of South Carolina, Columbia, SC 29208 USA; 5 Department of Mineral Sciences, Smithsonian Institution, Washington, DC 20013-7012 USA; 6 Beamline Science Division, Pohang Accelerator Laboratory, Pohang, 37673 Republic of Korea; 7 Graduate School of Semiconductor Materials and Devices Engineering, UNIST, Ulsan, 44919 Republic of Korea; 8 Hawaii Institute of Geophysics and Planetology, University of Hawaii at Manoa, 9700S Cass Ave, Argonne, IL 60439 USA; 9 Geochemical Research Center, Graduate School of Science, The University of Tokyo, Hongo 7-3-1, Bunkyo-ku, 113-0033 Tokyo, Japan; 10 Present Address: School of Earth Sciences and Environmental Engineering, GIST, Gwangju, 61005 Republic of Korea

**Keywords:** Mineralogy, Geochemistry

## Abstract

Manganese oxides are ubiquitous marine minerals which are redox sensitive. As major components of manganese nodules found on the ocean floor, birnessite and buserite have been known to be two distinct water-containing minerals with manganese octahedral interlayer separations of ~7 Å and ~10 Å, respectively. We show here that buserite is a super-hydrated birnessite formed near 5 km depth conditions. As one of the most hydrous minerals containing ca. 34.5 wt. % water, super-hydrated birnessite, i.e., buserite, remains stable up to ca. 70 km depth conditions, where it transforms into manganite by releasing ca. 24.3 wt. % water. Subsequent transformations to hausmannite and pyrochroite occur near 100 km and 120 km depths, respectively, concomitant with a progressive reduction of Mn^4+^ to Mn^2+^. Our work forwards an abiotic geochemical cycle of manganese minerals in subduction and/or other aqueous terrestrial environments, with implications for water storage and cycling, and the redox capacity of the region.

## Abstract (web summary)

The enigmatic relationship of birnessite and buserite, the two most representative phases in submarine nodules, has been established to reveal that buserite is a super-hydrated form of birnessite, forming at shallow terrestrial depth in the presence of water

## Introduction

Manganese (Mn) is the 3rd most abundant transition metal in the Earth’s crust^1^ and highly sensitive to redox processes indicative of specific aqueous environments^2,3^. While Earth’s crust has an average Mn abundance of 0.019 mol/kg^4^, concentrated Mn exists in the form of oxide nodules in oceanic sediments, which are found at almost all depths and latitude^2^. More than 30 different phases of Mn oxide/hydroxide minerals are known to date^2^ with the most dominant minerals being hydrogenetic vernadite (δ-MnO_2_), todorokite ((Ca,Na,K)_x_(Mn^4+^,Mn^3+^)_6_O_12_ ∙ H_2_O), buserite (Na_0.54_Mn_2_O_4_ ∙ 5.6(1)H_2_O) and birnessite (Na_0.54_Mn_2_O_4_ ∙ 1.5(1)H_2_O)^2,5^. The latter two are the major Mn oxide phases in diagenetic nodules, which account for ~60% of the chemical inventory of the Clarion Clipperton Zone nodules in the Pacific Ocean^6,7^ and contain billions of tons of other transition metals (Ni, Cu, Co, Li, Mo, Zr), as well as rare earth metals^5,8^. Although both phases are composed of the MnO_6_ octahedral layers but with different amounts of intercalated water molecules, their paragenetic relationship and relative stabilities have not been established yet, especially under the deep ocean and/or shallow terrestrial environments.

Compared to the Earth’s terrestrial environments, Martian basalts contain a higher MnO content of ~0.4 weight-% (wt. %)^9^. Recent observations from the Curiosity rover reports even 1–2 orders of magnitude higher concentrations of Mn in some rocks and emphasize the role of Mn minerals to be used as proxies to understand the evolution of the planet’s past aqueous environments^10,11^. Furthermore, the observation of dark Mn-rich rock surfaces is consistent with the presence of Mn oxide phases like birnessite^12^. There is strong evidence that such hydrous Mn oxides could have formed by recurring processes of strongly oxidizing conditions during past aqueous periods^13^.

In parallel to the diverse oxidation environments on the terrestrial surface, sub-surface regions are also controlled by spatial and secular variations of oxygen fugacity depending on the input and/or fractionation of oxidized sources. For example, magmas generated in the subducting arcs are typically more oxidized than those from mid-ocean ridge basalts^14^. Redox-sensitive elements with sufficient abundance in the mantle lithologies, such as Fe, H, C, and S, have been extensively studied to understand their geochemical cycle and influence on the redox state of the region^15^. Although Mn is ubiquitous in oceanic sediments and uniquely sensitive to high potential oxidants^16^, its redox cycle and role in the subsurface terrestrial environment, especially in the aqueous region where subducting fluid exists and interacts with an overriding mantle wedge, has not yet been explored. Although limited, previous studies suggested the hydrothermal origin of Mn found in the ridge or back-arc basin^17,18^ to be related to the subduction process^18–20^, as well as sedimentary Mn oxides to play a role in the forearc^21^ and in the arc regions^22^ based on the observed high *ε*^205^Tl (Thallium) values. Furthermore, Mn ores found in the accretionary belts along the Japan trench point to their origin from manganese nodules in the deep sea^23^, indicating subduction-related cycling of various Mn minerals.

In order to understand the stabilities and phase relationships of manganese oxide minerals under aqueous sub-surface environments, we have investigated the structural changes of birnessite in water at moderate pressures and temperatures up to ca. 4.37(1) GPa and 525(3) °C, thereby following a cold subduction geotherm^24^. We have observed super-hydration of birnessite to buserite at 0.19(2) GPa, equivalent to conditions found near 5 km depth, and subsequent transformations of buserite first to manganite at 2.33(1) GPa and 188(6) °C, which are conditions found near 70 km depth, then to hausmannite at 3.09(2) GPa and 314(3) °C, i.e., conditions found at 100 km depth, and then to pyrochroite at 3.88(1) GPa and 396(4) °C, i.e., conditions found near 120 km depth. During the course of the successive pressure and temperature-dependent transformations, a progressive reduction of Mn^4+^ to Mn^2+ ^occurs. Our observation sheds new light into the redox-related cycling of manganese oxide minerals and their impact on water transport and geochemistry in the aqueous sub-surface environments.

## Results and Discussion

### Super-hydration of birnessite at shallow depths and its relationship to buserite

The layered structure of birnessite is made of sheets of edge-sharing MnO_6_ octahedra separated by ~7 Å. Between these sheets water molecules and exchangeable alkaline and alkali-earth cations are located^25–27^. On the other hand, buserite with a ~10 Å interlayer spacing is known to be stable only under aqueous conditions^28–30^ and dehydrates into birnessite when present in a low humidity environment^25^. Although certain synthetic cation forms of buserite are reported to have two layers of water^31,32^, no established structural model exists for buserite.

**Fig. 1:**
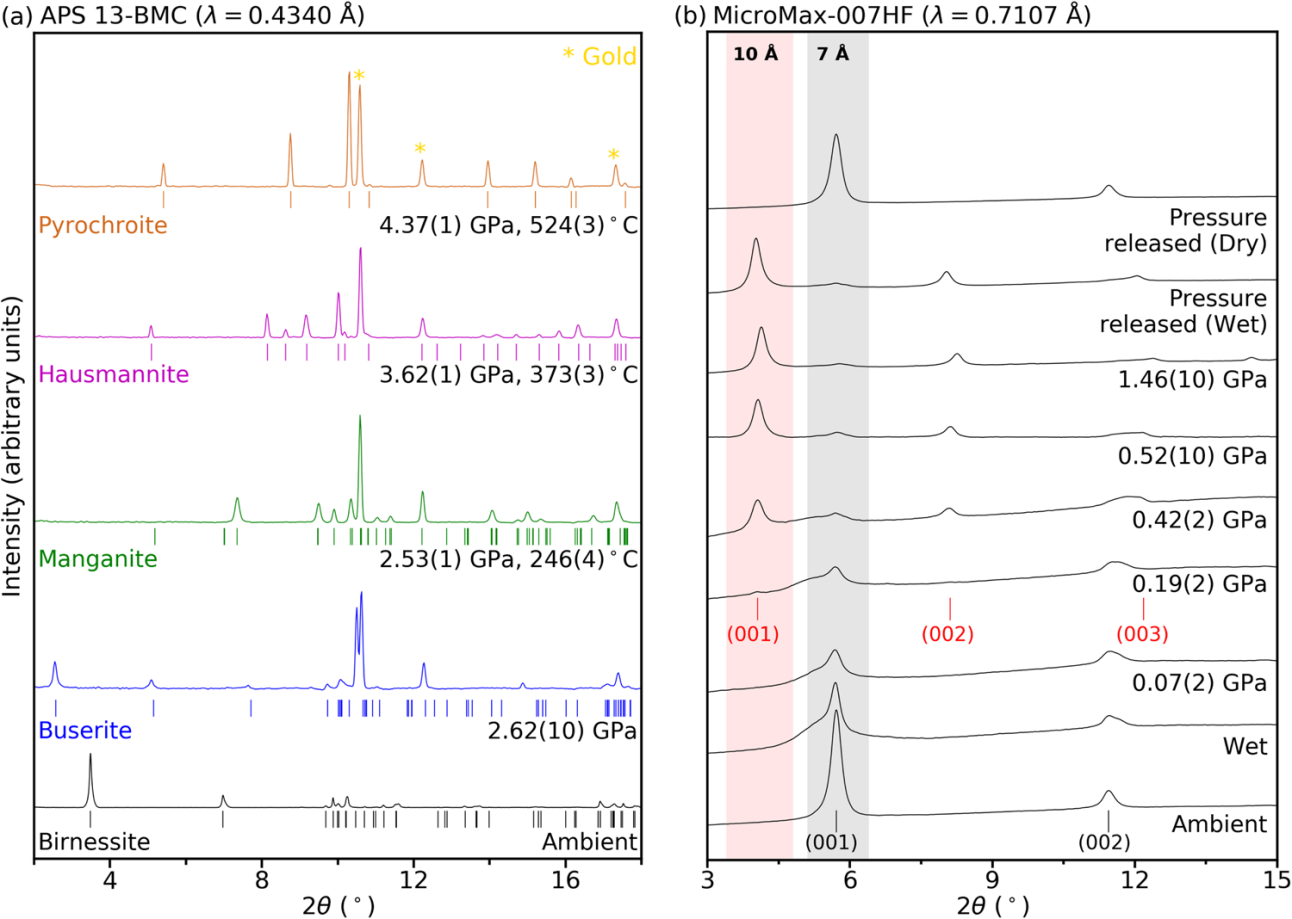
Synchrotron X-ray diffraction patterns showing successive transformations from birnessite. **a** Selected XRD patterns measured in-situ high-pressure conditions in water medium at the 13-BM-C beamline at APS. **b** Super-hydration of birnessite into buserite in a kilobar pressure regime. ‘Wet’ and ‘Dry’ indicate two different sample conditions at ambient pressure in water and after exposure to atmospheric humidity, respectively.

**Fig. 2:**
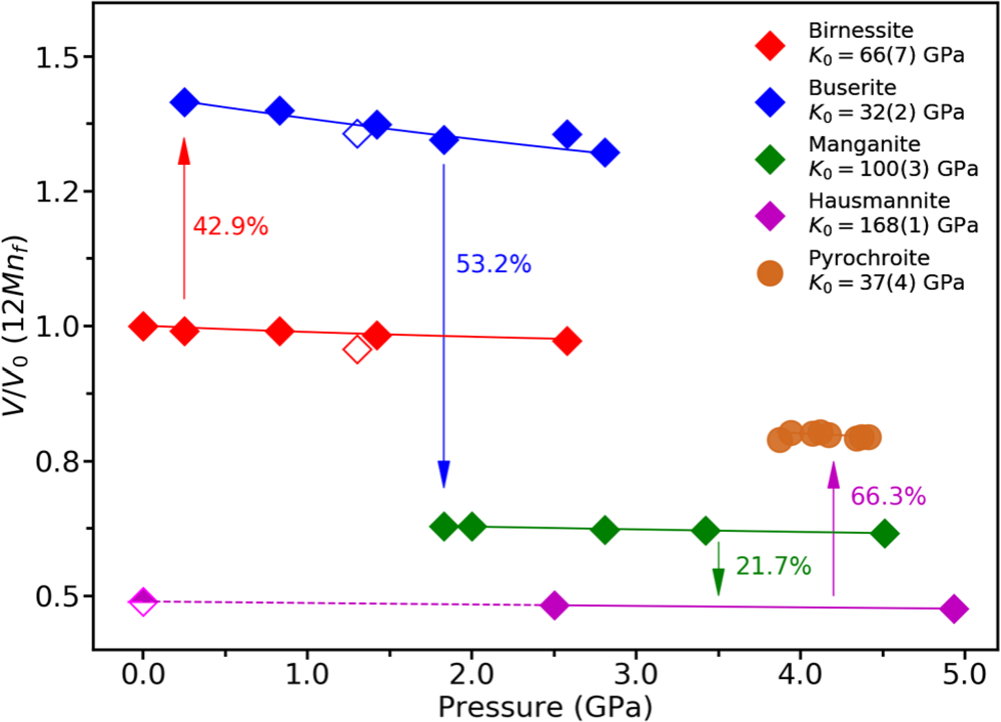
Changes in the normalized unit-cell volumes of birnessite and successive transformation products as a function of pressure. Unit cell volumes were normalized per 12 manganese atoms (12Mn_*f*_). Open symbols for birnessite and buserite are the data measured at the 6D beamline at PLS-II that have been used for the Rietveld refinements, and the circle symbols for pyrochroite were measured at the 13-BM-C beamline at APS. The half-filled symbol for hausmannite is after pressure release.

In-situ synchrotron X-ray powder diffraction (XRD) experiments were performed on synthetic birnessite (Na_0.54_Mn_2_O_4_ ∙ 1.5(1)H_2_O) in water using a resistively-heated diamond-anvil cell (RH-DAC) to follow the subduction geotherm model W1300 of the Tonga trench^24^ where Mn-abundant oceanic sediments^18,22^ in the Penrhyn Basin subduct^5^. Upon contact with water at ambient pressure, a shoulder peak appears at the low-angle side of the initial ~7 Å (001) X-ray diffraction peak, which is likely due to an increased turbostratic stacking disorder of the MnO_2_ layers^33^. Upon increase of pressure from 0.07(2) to 0.19(2) GPa, a new X-ray diffraction peak near ~10 Å d-spacing starts to form with a concomitant decrease in the intensity of the initial ~7 Å d-spacing (Fig. 1). At 0.42(2) GPa, the newly formed ~10 Å phase becomes dominant to the initial ~7 Å phase and persists up to 1.46(10) GPa at room temperature. The ~10 Å phase is recovered after pressure release when immersed in water. It transforms back to the initial ~7 Å phase, i.e., in a low humidity environment (Fig. 1b). The XRD pattern of the newly formed ~10 Å phase is indexed as a birnessite-related phase with the *c*-axis expanded by ca. 41.9 %, resulting in a volume expansion by ca. 42.9 % (Fig. 2 and Supplementary Table 1).

**Fig. 3:**
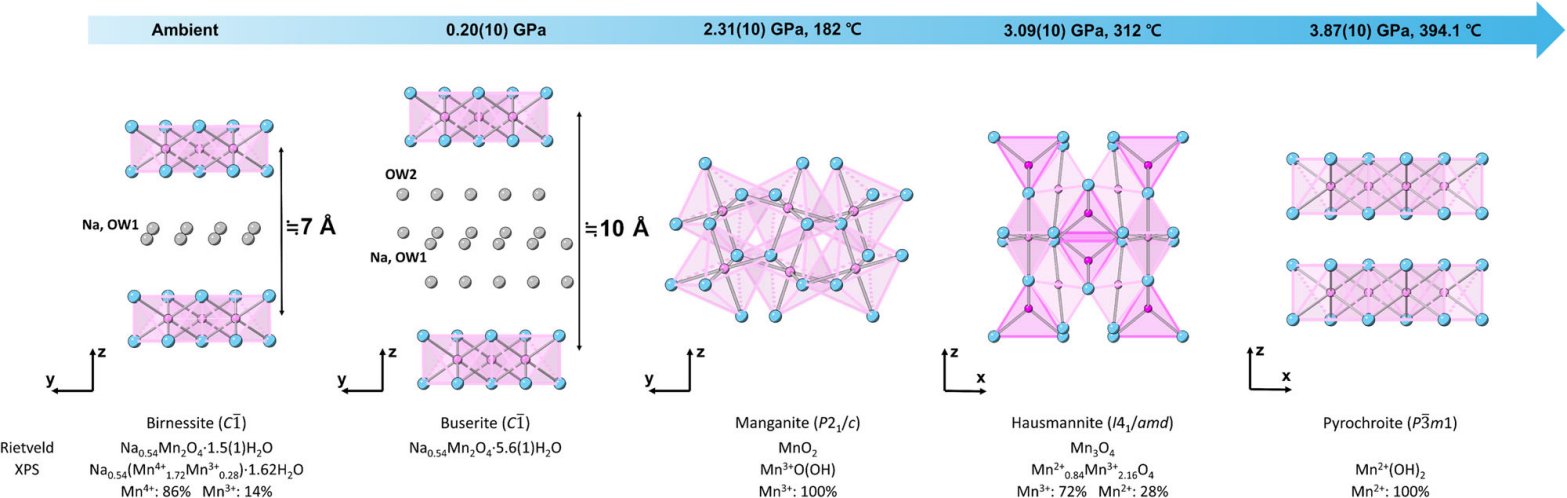
Successive transformations from birnessite to buserite, manganite, hausmannite, and pyrochroite along the simulated cold subduction geotherm conditions. Pink and light-blue spheres represent manganese and oxygen atoms, respectively. Gray spheres represent statistically disordered water molecules and sodium cations.

The structural models of the original birnessite and its expanded phase were refined using the Rietveld method of XRD data measured at ambient conditions and at 1.30(10) GPa, respectively. In the case of birnessite, a layer of statistically disordered Na^+^ cations and water molecules are located halfway between the MnO_6_ layers with an interlayer spacing of ~7 Å (Fig. 3), which is in accordance to the previously established model^23^. The Na^+^ content was fixed to 0.54 per unit cell, as determined by the X-ray photoelectron spectroscopy (XPS) analysis, which led to the refined chemical formula of Na_0.54_Mn_2_O_4_ ∙ 1.5(1)H_2_O (Supplementary Table 2). The refined water content of 1.5(1) H_2_O per unit cell is also in agreement with 1.62(1) H_2_O per unit cell obtained from the thermogravimetric analysis (TGA) (Supplementary Fig. 1). In the case of the expanded birnessite, Difference-Fourier analysis revealed two crystallographically distinct interlayer sites: the OW1 site in the middle of the layers and the OW2 site sandwiching the middle OW1 layer. The OW1 site was assumed to be occupied by statistically disordered Na^+^ cations and water molecules, as observed in the structural model of the original birnessite at ambient conditions. The OW2 site was then modeled using an oxygen atom of the water molecule, which showed near full occupancy and hence was fixed to unity. This led to the refined chemical formula of the expanded birnessite to be Na_0.54_Mn_2_O_4_ ∙ 5.6(1)H_2_O (Fig. 3 and Supplementary Table 2), a super-hydrated phase that is about 3.7 times more hydrated than the original birnessite, placing it among the most hydrated known minerals with a water wt. % of 34.5. This is higher than the 29 wt. % of H_2_O in the super-hydrated kaolinite^34^ and even rivals to the 34.7 wt. % of OH in gibbsite (Al(OH)_3_)^35^. Based on the structural and compositional characteristics, we consider the super-hydrated birnessite to be buserite and have thus established its paragenetic relationship to natural birnessite.

### Successive structural transformations of buserite at deeper depths

**Fig. 4:**
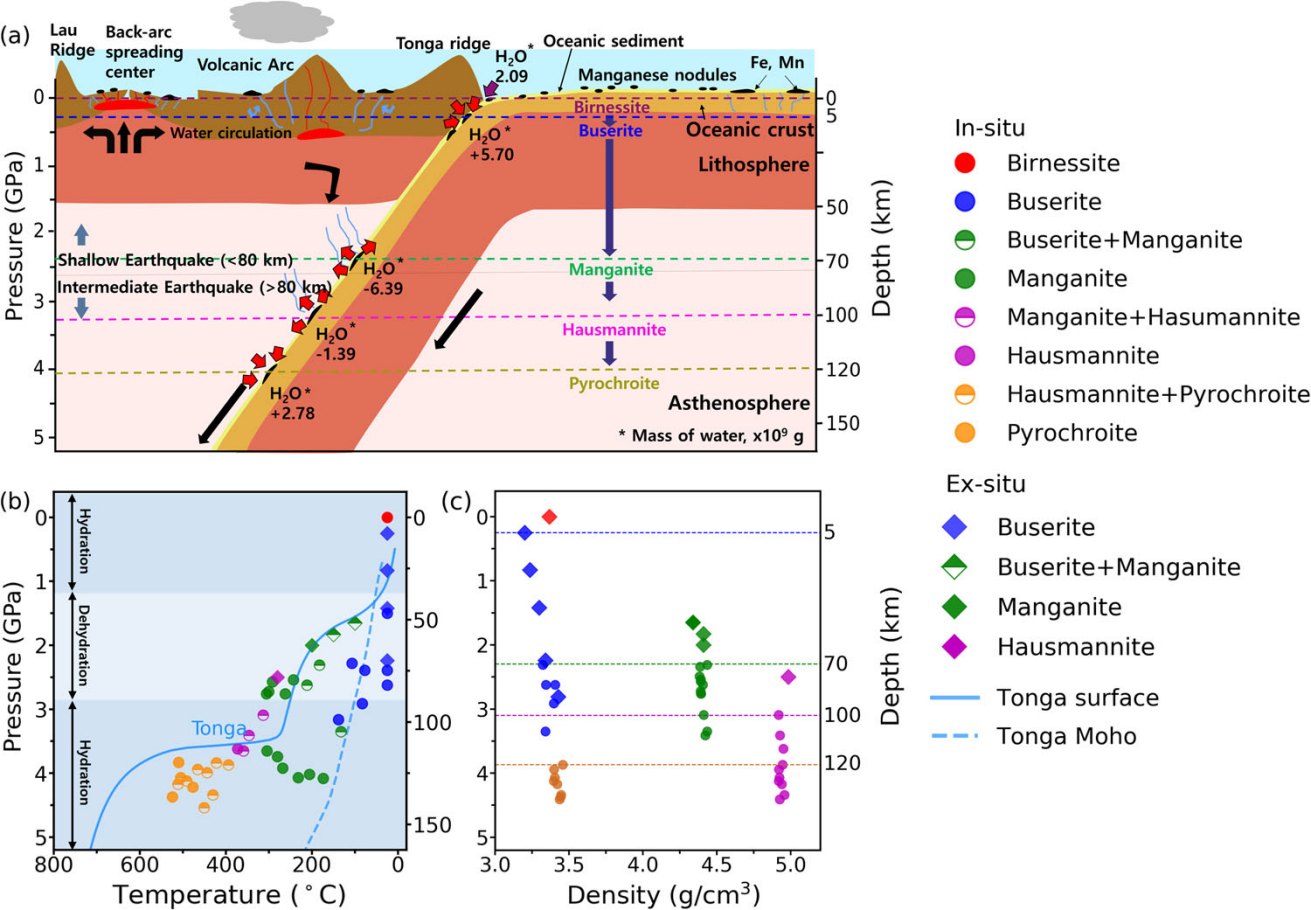
A proposed water transportation scheme by manganese oxide minerals along an aqueous cold subducting slab. **a** A schematic illustration of subducting birnessite to show the associated water transportation via super-hydration and subsequent transformations in the Tonga region. Mass of water is an uptake (+) from or release (−) into the surroundings (Supplementary Table 5). **b** Observed phase stabilities of manganese oxide minerals along the Tonga subduction geotherm following the model by Syracuse et al.^24^. **c** Depth-dependent changes in the crystal density (g/cm^3^) of manganese oxide minerals observed in our experiments.

The pressure where super-hydrated birnessite, i.e., buserite, starts to form, i.e., 0.19(2) GPa, corresponds to about 5 km depth along the cold subduction geotherm. Buserite, carrying ca. 34.5 wt.% water, remains stable upon increase in pressure and temperature to ca. 2.33(2) GPa and 188(6) °C, conditions found at ca. 70 km depth, where it transforms to manganite (ideally, Mn^3+^O(OH)) (Fig. 1a and Supplementary Fig. 2). Further increases in pressure and temperature lead to successive transformations from manganite to hausmannite (ideally, Mn^2+^ Mn^3+^_2_O_4_) at ca. 3.09(2) GPa and 314(3) °C, i.e., found near 100 km depth, and then to pyrochroite (ideally, Mn^2+^(OH)_2_) at ca. 3.88(1) GPa and 396(4) °C, i.e., conditions found near 120 km depth along the cold subduction geotherm (Figs. 1a, 3, 4 and Supplementary Fig. 2).

The structures of manganite, hausmannite, and pyrochroite have also been confirmed by the Rietveld refinements (Fig. 3 and Supplementary Tables 2–3). The crystalline quality and long-range order observed in these phases are remarkable, indicating no pressure-induced structural or chemical disorder (Fig. 1a). Unlike the super-hydration of birnessite to buserite, the transformation from buserite to manganite is reconstructive, i.e., irreversible, from the viewpoints of both structural and chemical changes in the connectivity of manganese oxide/hydroxide octahedral units and the release of Na^+^ cations and water molecules into the surroundings. The latter has been confirmed by micro X-ray fluorescence (XRF) elemental mapping of the recovered manganite sample formed at 2.29(10) GPa and 200 °C, which revealed substantial Na distribution around the gasket rims while Mn is uniformly distributed within the sample region (Supplementary Fig. 3).

Interestingly, the depth-dependent transformations from buserite to manganite, hausmannite, and pyrochroite are accompanied by a progressive reduction of Mn^4+^ to Mn^2+^. We have confirmed the sequential Mn reduction by measuring the oxidation states of respective recovered samples using XPS (Supplementary Figs. 4 and 5). For the original birnessite sample, the Mn^4+^ and Mn^3+^ contents are refined to ca. 86(11)% and 14(11)%, respectively, which are close to the previously reported values of 89.8% (Mn^4+^) and 10.2 % (Mn^3+^) in a triclinic Na-birnessite^36^ (Supplementary Figs. 4 and 5). Such a distribution of oxidation states would be upheld during the super-hydration of birnessite to buserite as it is mainly a water intercalation. In the recovered manganite sample, XPS shows the existence of only Mn^3+^, suggesting a selective reduction of Mn^4+^ in buserite during its transition to manganite (Supplementary Figs. 4 and 5). Hausmannite possesses a spinel structure with Mn^2+^ in the tetrahedral and Mn^3+^ in the octahedral sites in a 1:2 molar ratio (Fig. 3). Our XPS results confirm such an oxidation state for manganese with ca. 28(5)% for Mn^2+^ and 72(5)% for Mn^3+^, revealing a partial reduction of Mn^3+^ to Mn^2+^ during the reconstructive phase transition from manganite to hausmannite (Supplementary Figs. 4 and 5). On the other hand, pyrochroite adopts a CdI_2_-type structure with Mn^2+^(OH)_6_ octahedral sheets stacked along the *c*-axis (Fig. 3). Our XPS analyses show the existence of only Mn^2+^ in the recovered pyrochroite sample, suggesting complete reduction of Mn^3+^ to Mn^2+^ occurred during the transition from hausmannite to pyrochroite (Supplementary Figs. 4 and 5).

To verity the XPS analyses, bond valance sums (BVS) were calculated based on the derived structural models^37^. The presence of structural hydroxyl groups in manganite is corroborated by the significantly lower BVS of 1.26 and 1.38 for the two refined oxygen positions in the model (Supplementary Table 2). On the other hand, the separation of Mn^2+^ and Mn^3+^ sites in hausmannite results in different BVS values of 1.90 for the tetrahedral Mn^2+^ and 3.29 for the octahedral Mn^3+^ cations (Supplementary Table 2). For pyrochroite, reduction of the remaining Mn^3+^ to Mn^2+^ can be verified by its low BVS value of 1.69 (Supplementary Table 2). The BVS value of 2.12 for oxygen in hausmannite confirms the phase as an oxide, whereas significantly lower values of oxygen BVS’s are found in manganite and pyrochroite, in which a half and all of the oxygen anions exist as hydroxyl groups, respectively (Supplementary Table 2). In addition, the recovered manganite, hausmannite, and pyrochroite samples, when imaged by SEM, show different crystal morphologies, representing respective crystallo-chemical characteristics (Supplementary Fig. 4).

To gain insights into the stabilities of the manganese oxide phases formed along the cold subduction geotherm, pressure-volume data measured ex-situ at room temperature were fitted using the 2nd order Birch-Murgnahan equation of states^38^. Buserite is found to be about twice as compressible than the original birnessite: i.e., bulk modulus, *K*_*0*_, of birnessite is 66(7) GPa whereas *K*_*0*_ of buserite is 32(2) GPa (Fig. 2), which is comparable to that of NaCl-B2^39^. Such a compressible buserite then transforms into successively denser phases, namely manganite and hausmannite. Although limited in the pressure range, the derived bulk modulus of manganite is 100(3) GPa, which is ca. three times less compressible than buserite. Hausmannite, with a derived bulk modulus of 168(1) GPa, is identified as the least compressible and most dense phase in the transformation sequence observed in our study (Fig. 2 and Supplementary Table 4). Interestingly, the further transformation of hausmannite at conditions found near depths of 120 km led to the formation of the less dense and more compressible mineral, pyrochroite, with a derived bulk modulus of 37(4) GPa and a calculated density of 3.46 g/cm^3^, which are similar to the values found in buserite (Fig. 2 and Supplementary Table 4). We expect yet further transformations would be induced at deeper depths accompanied by a possible release of water by up to ca. 20.1 wt.% (Supplementary Table 5). It is important to note that, under a non-penetrating silicone oil medium, no structural transformations are observed, and the original birnessite persists up to 5.18(10) GPa with a bulk modulus of 60(1) GPa, which is essentially the same as the one determined from the experiments using penetrating water medium (Fig. 2 and Supplementary Fig. 6).

### Manganese oxides in aqueous terrestrial depth environments

It has long been proposed that the distribution of terrestrial manganese deposits is of hydrothermal origin and might be related to subduction processes^20^. Burns^40^ predicted manganese mineralization associated with subduction at the Tonga-Kermadec ridge, where manganese deposits of presumed hydrothermal origin are found over a distance of 1800 km^20^. Mineralization of manganese deposits has also been proposed to be contemporaneous with copper and other metals within calc-alkaline terrains associated with active and recently active subduction zones^19^. Subducting manganese minerals might be incorporated into fluids or melts which rise to form calc-alkaline hydrothermal vents or magmatism. Plank and Langmuir^41^ reported MnO content of approximately 2 wt.% in the sediment near the Tonga trench, and Rea and Ruff^42^ estimated an average sediment flux through the Tonga trench by ca. 164.7 × 10^12 ^g/yr. If we assume that half of the subducting Mn would be in the form of birnessite, the amount of water transported via super-hydrated birnessite, i.e., buserite, along the Tonga subduction zone would be ca. 7.78 Gg/yr, which is about 1/7 of water that can be transported by super-hydrated kaolinite, i.e., ca. 57.08 Gg/yr^34^, assuming its abundance in the sediments to be ca. 10 wt.% (Supplementary Table 5). Such an estimation is in line with the high *ε*^205^Tl observed in the Tonga-Kermadec lava^22^ as Tl isotopes are known to fractionate only when sorbed onto birnessite^43^. It is also interesting to note that the depth range where the super-hydrated sedimentary minerals exist would span from ca. 5 km to 200 km along a cold subduction zone, i.e., super-hydrated birnessite between ca. 5 km and 70 km and super-hydrated kaolinite between ca. 75 km and 200 km. A schematic illustration of water transportation via subducting manganese minerals is shown in Fig. 4a.

The depth where super-hydrated birnessite, e.g., buserite, forms is as shallow as 5 km along a cold subduction zone^24^ (Fig. 4a). The fact that buserite, as the most hydrated mineral with ca. 34.5 water wt. %, forms at the interface between a subducting slab and an overriding wedge needs to be taken into account when modeling subduction-related processes, especially in such shallow depths region. It has recently been shown that the presence of low-friction layered minerals, such as smectite, may play an important role in dynamic weakening processes leading to large earthquakes^44,45^. The low shear strength of layered minerals with weak interlayer forces, particularly upon abrupt interlayer expansion via super-hydration, would promote earthquake ruptures by large slips in a shallow subduction interface. In fact, we showed that buserite is two times more compressible than birnessite and possesses an anisotropic compression behavior, which may contribute to oriented microstructures and seismic anisotropy (Supplementary Fig. 6).

Upon transformation of buserite into denser hydrous manganite, at ca. 70 km depth, a significant amount of water by ca. 24.3 wt.% is released together with a reduction of Mn^4+^ into Mn^3+^ and exfoliation of Na^+^ ions. This would increase the relative fluid volume in the region and affect the alkalinity of the subducting fluid^46^. Water release and reduction of Mn in such a depth range might trigger the release of the sorbed Tl to be incorporated to serpentinization in the Mariana forearc^21^. At a depth near 100 km, a further release of water by ca. 10.2 wt.% occurs due to the transformation of manganite into an anhydrous denser mineral, hausmannite (Fig. 4 and Supplementary Table 4). Although limited in abundance in the case of Mn oxides, such a dehydration and subsequent embrittlement into hard minerals along a subducting slab have been established to be associated with the formation of intermediate-depth earthquakes^47^, which account for ca. 86% of earthquakes down to ca. 150 km depth along the Tonga subduction zone. A further change in the alkalinity of surrounding fluid is anticipated as the transformation of manganite into hausmannite is accompanied by a partial reduction of ca. 1/3 Mn^3+^ into Mn^2+^. Conversely, subsequent transformation of anhydrous hausmannite into pyrochroite at a depth near ca. 120 km involves the incorporation of water, i.e., hydration, in the form of structural hydroxyls with ca. 20.1 wt.% (Fig. 4a and Supplementary Table 5). This is accompanied by a further reduction of Mn^3+^ in hausmannite to Mn^2+^, thereby completing the progressive depth-dependent manganese reduction sequence from Mn^4+^/Mn^3+^ to Mn^2+^.

Most of the dissolved manganese in the ocean are known to be in the form of Mn^2+^ in hydrothermal fluids^48–51^. As mentioned earlier, recently discovered high concentrations of Mn^2+^ in rocks in the Gale crater on Mars^10,11^ and in Martian meteorites^52,53^ point to past episodes of strongly oxidizing conditions and the presence of aqueous environments. Taking into account our observations of successive and progressive transformation of manganese oxide minerals as a function of depth may also guide understanding commonalities and differences of complex geophysical and geochemical processes between different terrestrial planets. Our results thus provide a new platform to understand the phase relationships of various manganese oxide minerals in an extended terrestrial depth environment to impact water transport, redox capacity, and related elemental cycling of the region.

## Methods

### Samples

A powder sample of synthetic birnessite was provided by Kyungpook National University^54^. The chemical composition of the sample was analyzed using X-ray photoelectron spectroscopy (XPS) at the Yonsei Center for Research Facilities and revealed a composition of (Na_0.54(2)_K_0.01(1)_)Mn_1.92(4)_O_4_ ∙ 1.62(1)H_2_O. The H_2_O content of birnessite was determined using thermogravimetric analysis (TGA, SDT Q600, TA Instruments) at the Industry University Cooperation Foundation at Hanyang University. During TGA, argon gas was flowing at 100 ml/min, and the temperature was increased to 850 °C at a rate of 10 °C/min (Supplementary Fig. 1). To confirm the phase purity, the sample was loaded into a borosilicate-capillary of 0.5 mm diameter and measured using a laboratory XRD up to 90˚ in 2*θ* (Rigaku MicroMax-007HF, Mo *K*_*α*_ radiation) at the Institute for High-pressure Mineral Physics and Chemistry at Yonsei University. The crystalline phase was identified as pure birnessite with unit-cell parameters of *a* = 5.174(1) Å, *b* = 2.854(1) Å, *c* = 7.321(1) Å, *α* = 89.56(3)˚, *β* = 103.19(2)˚, *γ* = 90.05(4)˚ in space group *C*$\bar{1}$, which are similar to values reported in the literature^25^.

### Ex-situ high-pressure synchrotron X-ray powder diffraction

Ex-situ high-pressure and temperature synchrotron X-ray powder diffraction (XRD) experiments were performed at the 3D beamline at the Pohang Light Source II (PLS-II) at the Pohang Accelerator Laboratory (PAL), Pohang, Korea. X-ray beam from a bending magnet was monochromatized using a Si(111) double-crystal to select a wavelength of 0.6888(1) Å (18 keV) and reduced to a 100 μm diameter using a pinhole. A MAR 345 image plate was used as a detector, which was calibrated using a NIST LaB_6_ standard (SRM-660C). A symmetric-type diamond anvil cell (DAC) containing a pair of type-I diamonds with 700 μm diameter culets was used to create hydrostatic pressures. A few small ruby spheres were added to the sample chamber as pressure markers in the fluorescence method^55^. Additional synchrotron X-ray diffraction experiments were performed at the 6D beamline of PLS-II using a Raynoix Mx225-HS detector to confirm the observations made at the 3D beamline and to obtain higher quality XRD patterns suitable for the Rietveld analysis. The X-ray used was 0.6530(1) Å in wavelength and 150 μm in diameter. We also performed supplementary XRD measurements at the BL-18C beamline at Photon Factory (PF), KEK using synchrotron X-ray of 20 keV and at the Institute for High-pressure Mineral Physics and Chemistry at Yonsei University using micro-focused rotating-anode X-ray from Mo target (Rigaku MicroMax-007HF).

Two different types of fluids were used as a pressure-transmitting medium (PTM): water as a pore-penetrating PTM to allow pressure-induced hydration (i.e., super-hydration^56^) (Supplementary Fig. 2a) and silicone oil as a nonpore-penetrating PTM to probe the intrinsic compressibility of the sample under quasi-hydrostatic conditions (Supplementary Fig. 6). The maximum pressure applied was 4.9(1) GPa with water PTM and 5.2(1) GPa with silicone oil PTM. When using water PTM, occasional heating was done ex-situ by placing the DAC in an oven for 1 hr in order to melt the high-pressure ice formed and to simulate the cold subduction thermal gradient.

### In-situ high-pressure synchrotron X-ray powder diffraction

In-situ high-pressure and temperature synchrotron XRD experiments were performed using a resistively-heated DAC (RH-DAC) at the 13-BM-C beamline at the Advanced Photon Source at the Argonne National Laboratory. The X-ray beam used was monochromated to 0.4340 Å with eV bandwidth in wavelength, and a Pliatus3 1 M detector was used to collect the XRD patterns. The sample-to-detector distance and the tilt of the detector were calibrated with a NIST LaB_6_ powder standard. A membrane-driven pressure control system (GE Pace5000) was coupled to the RH-DAC, and a resistive heating coil (Kanthal wire) was installed around the anvil to create simultaneous high pressure and temperature conditions following the cold subduction thermal gradient^24^. The temperature was monitored using a K-type thermocouple attached to the anvil close to the sample chamber, which provided the maximum uncertainties of ±3 °C. A small amount of Au powder was mixed with the sample powder to be used as an internal pressure marker during the in-situ experiments^57^.

### High-pressure XRD in a kilobar regime using FluoSphere^®^

A FluoSpheres^**®**^ method^58^ was applied to investigate the onset pressure of super-hydration in a kilobar regime, i.e., pressures below ca. 0.25 GPa. For this ‘low-pressure’ experiment, a pair of type-I diamonds with 1000 μm diameter culets were used under water PTM. A green laser with 515 nm wavelength was used to excite the FluoSphere^**®**^ **(**and ruby as an independent pressure marker above ca. 0.25 GPa). Laboratory XRD data were measured from 0.007(20) GPa to a maximum pressure of 0.99(2) GPa at the Institute for High-pressure Mineral Physics and Chemistry at Yonsei University. At each pressure, the sample in the DAC was in equilibrated for at least 5 min before the XRD measurement (Fig. 1b).

### Rietveld structure refinements

The structure refinements of the original birnessite and its super-hydration and transformation products, i.e., buserite, manganite, hausmannite, and pyrochroite, were performed using the Rietveld method implemented in the GSAS suite of programs^59,60^ (Supplementary Table 2). For the birnessite and buserite models, the background was estimated manually, while for manganite, hausmannite, and pyrochroite, the background was fitted using a Chebyshev polynomial with ≤34 coefficients. Peak profiles were fitted using the Pseudo-Voigt profile function proposed by Thompson et.al^61^. with the asymmetry correction introduced by Finger et al.^62^. Except for buserite, previously determined crystal structures were used as starting models for the respective refinements^25,63–65^. In the case of buserite, Difference-Fourier synthesis was performed after initial refinements using only the MnO_2_ layers, which revealed residual electron densities at two crystallographically distinct interlayer sites: the OW1 site in the middle of the MnO_2_ layers at (−0.038(1), 0.304(1), 0.477(1)) was modeled as an oxygen-sodium mixed site, as in the birnessite model, while the OW2 site at (−0.169(5), 0.825(3), 0.320(1)), sandwiching the OW1 site in the middle, was modeled as an oxygen atom of a water molecule. The occupancy of Na at the OW1 site was fixed to the value in the birnessite model, i.e., 0.54 per unit cell, while the occupancy of O at the OW2 site was fixed to unity as the refinement showed nearly full occupancy. Soft restraints were applied to the Mn-O interatomic distances for the MnO_6_ octahedron, e.g., 1.93(1) Å^25^, to supplement the observed data. In order to further reduce the number of parameters, the atomic displacements were modeled to be isotropic (U_iso_) and grouped to be the same for all the atoms. The final convergence was achieved by refining the atomic fractional occupancy of oxygen at the OW1 site, displacement parameters, 2*θ* zero, profile parameters, scale factor, and lattice parameters. To derive the bulk moduli and linear compressibilities, the LeBail profile fitting was performed for all ex-situ XRD patterns (Fig. 2 and Supplementary Table 1), and the 2nd order Birch-Murgnahan equation of states was used to fit the volume-pressure data (Supplementary Fig. 6). Final structural models from the Rietveld refinements are summarized in Fig. 3 and Supplementary Tables 2–3 with the final fits shown in Supplementary Fig. 7. Bond valence sums (BVS) were calculated using the final refined models and the EXPO 2014 program^37^.

### Postmortem analyses of the recovered samples

Each recovered sample was characterized by XRD before proceeding with other measurements. Scanning electron microscopy (SEM) was performed to compare the crystal morphologies of the recovered manganite (from 2.50(10) GPa and 200 °C), hausmannite (from 3.70(10) GPa and 300 °C), and pyrochroite (from 4.37(1) GPa and 525(3) °C) samples to that of the original birnessite. Each sample was coated with platinum, and a JEOL-7800F was used at 10 kV or 15 kV (Supplementary Fig. 4). XPS was employed to determine the oxidation states of manganese in the recovered manganite (from 1.98(10) GPa and 200 °C), hausmannite (from 2.50(10) GPa and 300 °C), and pyrochroite (from 4.37(1) GPa and 525(3) °C) samples. The X-ray source used was an Al Kα at pass energy 40 eV and step size 0.1 eV (K-alpha, Thermo VG), and the samples were measured under vacuum conditions (Supplementary Figs. 4). In order to observe the transformation pathways and products via atomistic scale imaging, scanning transmission electron microscopy (STEM) was performed on the recovered manganite (from 4.20(10) GPa and 200 °C) and hausmannite (from 6.92(10) GPa and 300 °C) samples using a JEOL JSM 5610 LV at Yonsei University and a JEOL JEM-2100F equipped with a CEOS C_s_ corrector on the illumination system at the University of South Carolina. The images were recorded with a 200 kV electron beam and a Fischione Model 3000 HAADF detector (Supplementary Fig. 8). The extraction of Na^+^ cations during the transformation of buserite to manganite was confirmed by X-ray fluorescence (XRF) spectroscopy using the recovered manganite sample from 2.29(10) GPa and 200 °C using the M4 Tornado instrument (Bruker Co.) at the Institute for High-pressure Mineral Physics and Chemistry at Yonsei University. A 2D elemental distribution was measured using a 25 μm diameter X-ray beam from an Rh-target (Supplementary Fig. 3).

## Supplementary information

Supplementary Information

## Data Availability

All data generated or analyzed during this study are included with this published article and its Supplementary Information.
