# Supplementary material for: Super-hydration and reduction of manganese oxide minerals at shallow terrestrial depths
Source: Nat Commun. 2022 Apr 11;13:1942. doi: 10.1038/s41467-022-29328-y (PMC9001738; doi:10.1038/s41467-022-29328-y)
Supplement: Supplementary file 1 — Supplementary Information [file 41467_2022_29328_MOESM1_ESM.pdf]

Supplementary Information

Super-hydration and reduction of manganese oxide minerals at shallow terrestrial depths

Seohee Yun[1], Huijeong Hwang[1], Gilchan Hwang[1], Yeongkyoo Kim[2], Douglas Blom[3], Thomas Vogt[4], Jeffrey E. Post[5], Tae-Yeol Jeon[6], Tae Joo Shin[7], Dong-Zhou Zhang[8], Hiroyuki Kagi[9], Yongjae Lee[1,*]

[1]Department of Earth System Sciences, Yonsei University, Seoul 03722, Republic of Korea. [2]School of Earth System Sciences, Kyungpook National University, Daegu, 41566, Republic of Korea. [3]Nano Center and Department of Chemical Engineering, University of South Carolina, Columbia, South Carolina 29208, USA. [4]Nano Center, Departments of Chemistry & Biochemistry and Chemical Engineering, University of South Carolina, Columbia, South Carolina 29208, USA. [5]Department of Mineral Sciences, Smithsonian Institution, Washington, D.C. 20013-7012, USA. [6]Beamline Science Division, Pohang Accelerator Laboratory, Pohang 37673, Republic of Korea, [7]UNIST Central Research Facilities & School of Natural Science UNIST, Ulsan 44919, Republic of Korea, [8]Hawaii Institute of Geophysics and Planetology, University of Hawaii at Manoa, 9700 S Cass Ave, Argonne, IL 60439, USA, [9]Geochemical Research Center, Graduate School of Science, The University of Tokyo, Hongo 7-3-1, Bunkyo-ku, 113-0033 Tokyo, Japan

Correspondence to yongjaelee@yonsei.ac.kr

Supplementary Table 1. Unit cell parameters and volume of birnessite and its super-hydration and transformation products, buserite, manganite, and hausmannite, as a function of increasing pressure and heat treatment under water medium.

**Pressure (GPa)**	**Temperature (°C)**	**Mineral**	***a*** **(Å)**	***b*** **(Å)**	***c*** **(Å)**	***α*** **(°)**	***β*** **(°)**	***γ*** **(°)**	**Unit cell volume (Å^3)**
Ambient	25	Birnessite	5.179(1)	2.850(1)	7.324(1)	89.48(2)	103.24(2)	89.78(2)	105.24(2)
0.25(10)	25	Birnessite	5.169(7)	2.841(3)	7.323(4)	89.00(20)	103.90(10)	89.10(20)	104.30(10)
		Buserite	5.159(5)	2.860(2)	10.393(5)	88.99(8)	103.66(9)	90.2(2)	149.00(12)
0.83(10)	25	Birnessite	5.176(8)	2.840(3)	7.266(5)	89.5(1)	103.3(1)	89.9(2)	103.97(10)
		Buserite	5.145(4)	2.862(3)	10.299(4)	89.5(2)	103.6(8)	90.2(4)	147.40(10)
1.42(10)	25	Birnessite	5.171(32)	2.837(7)	7.252(11)	89.7(3)	103.4(3)	89.9(5)	103.54(49)
		Buserite	5.138(3)	2.842(2)	103.202(2)	89.8(1)	103.9(1)	91.0(1)	144.62(9)
2.24(10)	25	Birnessite	5.153(6)	2.827(7)	7.225(10)	89.7(2)	103.5(3)	90.3(5)	102.35(29)
		Buserite	5.121(4)	2.845(3)	10.090((3)	90.7(2)	103.8(1)	90.2(1)	142.77(18)
1.83(10)	150	Birnessite	5.149(2)	2.828(1)	7.215(3)	89.6(1)	103.2(1)	90.1(1)	102.26(5)
		Buserite	5.100(2)	2.841(2)	10.061(2)	89.6(1)	103.7(1)	90.2(1)	141.60(7)
		Manganite	5.275(2)	5.226(2)	5.274(1)	90	114.3(1)	90	132.53(1)
2.81(10)	25	Birnessite	5.157(4)	2.823(3)	7.151(8)	89.5(1)	103.2(1)	89.8(2)	101.35(16)
		Buserite	5.091(3)	2.830(1)	9.944(2)	89.5(1)	103.7(1)	90.2(1)	139.17(7)
		Manganite	5.254(1)	5.206(1)	5.257(1)	90	114.2(1)	90	131.15(1)
2.00(10)	200	Manganite	5.269(1)	5.230(1)	5.270(1)	90	114.2(1)	90	132.50(1)
3.42(10)	25	Manganite	5.235(1)	5.190(1)	5.234(1)	90	114.1(1)	90	129.83(1)
2.50(10)	280	Hausmannite	5.760(1)	5.760(1)	9.194(3)	90	90	90	305.05(1)
4.93(10)	25	Hausmannite	5.741(1)	5.741(1)	9.134(1)	90	90	90	301.07(5)
Release	25	Hausmannite	5.795(1)	5.795(1)	9.215(2)	90	90	90	309.50(2)

Supplementary Table 2. Rietveld structural models of birnessite, buserite, manganite, hausmannite, and pyrochroite using synchrotron XRD patterns measured at different pressures (and temperature) under water medium.

Birnessite — Ambient, $C\bar{1}$

Atom	Wyckoff letter	Mutiplicity	Fractional Coordinate	
Mn (BVS 3.72v.u.)	*a*	2	x	0
			y	0
			z	0
			Occ	1
			Uiso	0.03(1)
O (BVS 1.86v.u.)	*i*	4	x	0.368(1)
			y	-0.003(1)
			z	0.131(1)
			Occ	1
			Uiso	0.03(1)
Na	*i*	4	x	-0.046(5)
			y	0.296(5)
			z	0.469(5)
			Occ	0.135
			Uiso	0.03(1)
OW	*i*	4	x	-0.046(5)
			y	0.296(5)
			z	0.469(5)
			Occ	0.38(2)
			Uiso	0.03(1)

Buserite — 1.30(10) GPa, $C\bar{1}$

Atom	Wyckoff letter	Mutiplicity	Fractional Coordinate	
Mn (BVS 3.71v.u.)	*a*	2	x	0
			y	0
			z	0
			Occ	1
			Uiso	0.051(2)
O (BVS 1.89v.u.)	*i*	4	x	0.371(1)
			y	-0.015(1)
			z	0.098(1)
			Occ	1
			Uiso	0.051(2)
Na	*h*	2	x	-0.038(1)
			y	0.304(1)
			z	0.477(1)
			Occ	0.135
			Uiso	0.051(2)
OW1	*h*	2	x	-0.038(1)
			y	0.304(1)
			z	0.477(1)
			Occ	0.399(6)
			Uiso	0.051(2)
OW2	*i*	4	x	-0.169(5)
			y	0.825(3)
			z	0.320(1)
			Occ	1
			Uiso	0.051(2)

Manganite — 3.42(10) GPa, $P2_1/c$

Atom	Wyckoff letter	Mutiplicity	Fractional Coordinate	
Mn (BVS 2.64v.u.)	*e*	4	x	-0.259(8)
			y	0.007(1)
			z	0.737(7)
			Occ	1
			Uiso	0.007(1)
O1 (BVS 1.26v.u.)	*e*	4	x	0.317(3)
			y	0.104(2)
			z	0.629(3)
			Occ	1
			Uiso	0.007(1)
O2 (BVS 1.38v.u.)	*e*	4	x	0.873(1)
			y	0.139(2)
			z	0.129(1)
			Occ	1
			Uiso	0.007(1)

Hausmannite — 4.93(10) GPa, $I4_1/amd$

Atom	Wyckoff letter	Mutiplicity	Fractional Coordinate	
Mn1 (BVS 3.29v.u.)	*d*	8	x	0
			y	0
			z	0.5
			Occ	1
			Uiso	0.018(1)
Mn2 (BVS 1.90v.u.)	*a*	4	x	0
			y	0.75
			z	0.125
			Occ	1
			Uiso	0.018(1)
O (BVS 2.12v.u.)	*h*	16	x	0
			y	0.464(1)
			z	0.255(1)
			Occ	1
			Uiso	0.018(1)

Pyrochroite* — 4.37(1) GPa, 524 °C, $P\bar{3}m1$

Atom	Wyckoff letter	Mutiplicity	Fractional Coordinate	
Mn (BVS 1.65v.u.)	*a*	1	x	0
			y	0
			z	0
			Occ	1
			Uiso	0.017(3)
O (BVS 0.83V.U.)	*d*	2	x	1/3
			y	2/3
			z	0.257(1)
			Occ	1
			Uiso	0.017(3)

* From *in-situ* data

Supplementary Table 3. Interatomic distances of the Rietveld refined models of birnessite, buserite, manganite, hausmannite, and pyrochroite.

**Birnessite Ambient**		**Buserite 1.30(10) GPa**		**Manganite 3.42(10) GPa**		**Hausmannite 4.93(10) GPa**		**Pyrochroite** 4.37(1) GPa, 524 ℃**	
Mn-O x2	1.93(1)	Mn-O x2	1.93(1)	Mn-O1	1.91(2)	Mn3+-O x4	1.89(1)	Mn-O x2	2.23(1)
Mn-O x2	1.93(1)	Mn-O x2	1.93(1)	Mn-O1	2.13(2)	Mn3+-O x2	2.25(1)	Mn-O x2	2.23(1)
Mn-O x2	1.93(1)	Mn-O x2	1.93(1)	Mn-O1	2.28(1)	Mean	2.01	Mn-O x2	2.23(1)
Mean	1.93	Mean	1.93	Mean	2.11	Mn2+-O x4	2.03(1)	Mean	2.23
O-OW (Na)	2.47(1)	O-OW2	2.55(1)	Mn-O2	2.00(1)	Mean	2.03		
O-OW (Na)	2.93(1)	O-OW2	2.88(1)	Mn-O2	2.01(1)				
OW-OW	2.28(1)	O-OW2	2.90(1)	Mn-O2	2.12(1)				
OW-OW	2.85(1)	OW2-OW2	2.80(1)	Mean	2.04				
OW-OW	2.96(1)	OW2-OW2	2.92(1)						
		OW2-OW2	2.94(1)						
		Na-OW2	2.10(2)						
		Na-OW2	2.11(1)						
		Na-OW2	2.13(1)						
		Na-OW2	2.77(1)						
		Na-Na	*1.25(1)						
		Na-Na	*1.76(1)						
		Na-OW1	2.36(1)						
		Na-OW1	2.80(1)						
		Na-OW1	2.90(1)						
		Na-OW1	2.92(1)						
		Na-OW1	2.94(1)						

* Simultaneous occupancy excluded

****** *In-situ* data.

Supplementary Table 4. Changes in the crystal densities upon the super-hydration of birnessite into buserite and subsequent transformations into manganite, hausmannite, and pyrochroite.

**Pressure (GPa)**	**Temperature (K)**	**Mineral**	**Density (g/cm^3)**
Ambient*	298	Birnessite	3.37
2.62	298	Buserite	3.34
2.91	356	Buserite	3.40
3.35	405	Buserite	3.34
		Manganite	4.43
2.62	484	Buserite	3.41
		Manganite	4.41
2.54	516	Manganite	4.39
2.72	574	Manganite	4.39
3.09	586	Manganite	4.41
		Hausmannite	4.92
3.41	620	Manganite	4.42
		Hausmannite	4.93
3.62	646	Hausmannite	4.95
3.87	667	Hausmannite	4.95
		Pyrochroite	3.46
3.94	739	Hausmannite	4.92
		Pyrochroite	3.40
4.07	778	Hausmannite	4.92
		Pyrochroite	3.40
4.17	797	Pyrochroite	3.44

* Data from PLS 3D experiments.

Supplementary Table 5. Estimated amounts of water that can be transported by manganese oxide minerals studied here, assuming an average sediment flux through the Tonga trench by ca. 164.7 x 10^{12} g/yr[1], MnO content of approximately 2 wt. %[2] in the sediment near the Tonga trench, and half of the subducting Mn is in the form of birnessite. Similar calculation has been applied for super-hydrated kaolinite assuming its abundance by 10 wt. % in the Tonga sediment.

	Subduction amount (mol/year, 10^7)	Subduction amount of water (g/year, 10^9)	Uptake (+) or release (-) of water (mol/year, 10^7)	Uptake (+) or release (-) of water (g/year, 10^9)
Birnessite	7.72	2.09	31.66	5.70
H_2O in Birnessite (12.4 wt. %)	11.58			
Buserite	7.72	7.78		
H_2O in Buserite (34.5 wt. %)	43.24			
Manganite	15.44	1.39	-35.52	-6.39
H_2O in Manganite (10.2 wt. %)	7.72			
Hausmannite	5.15	0	-7.72	-1.39
H_2O in Hausmannite (0.00 wt. %)	0			
Pyrochroite	15.44	2.78	+15.44	+2.78
H_2O in Pyrochroite (20.1 wt. %)	15.44			
Kaolinite (10%)	6.38	22.97	+18.95	34.11
H_2O in Kaolinite	12.76			
Super-hydrated Kaolinite	6.38	57.08		
H_2O in Super-hydrated Kaolinite	31.71			

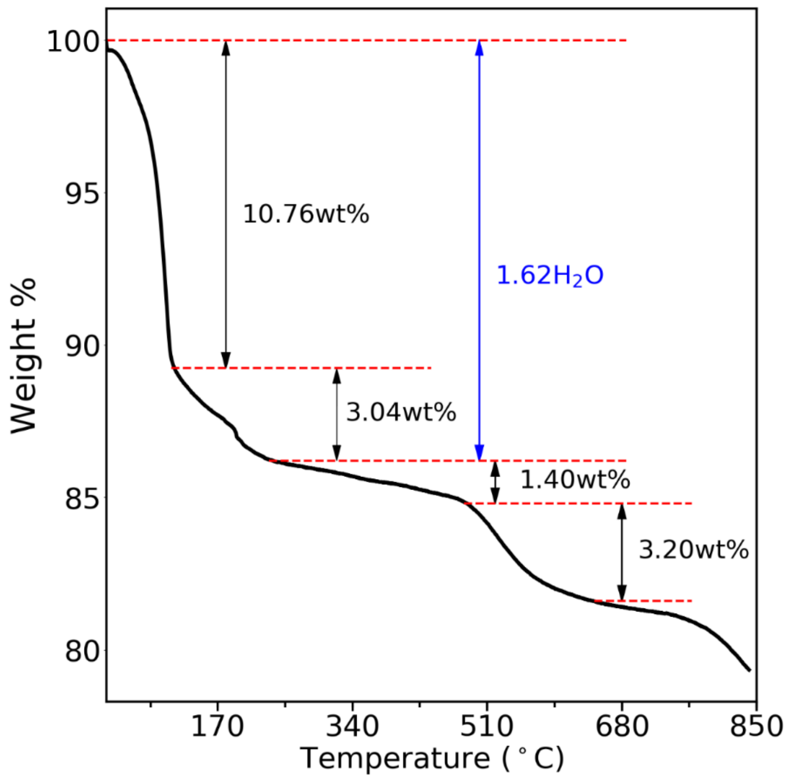

Supplementary Figure 1. Thermal-gravimetric analysis of the original Na-birnessite.

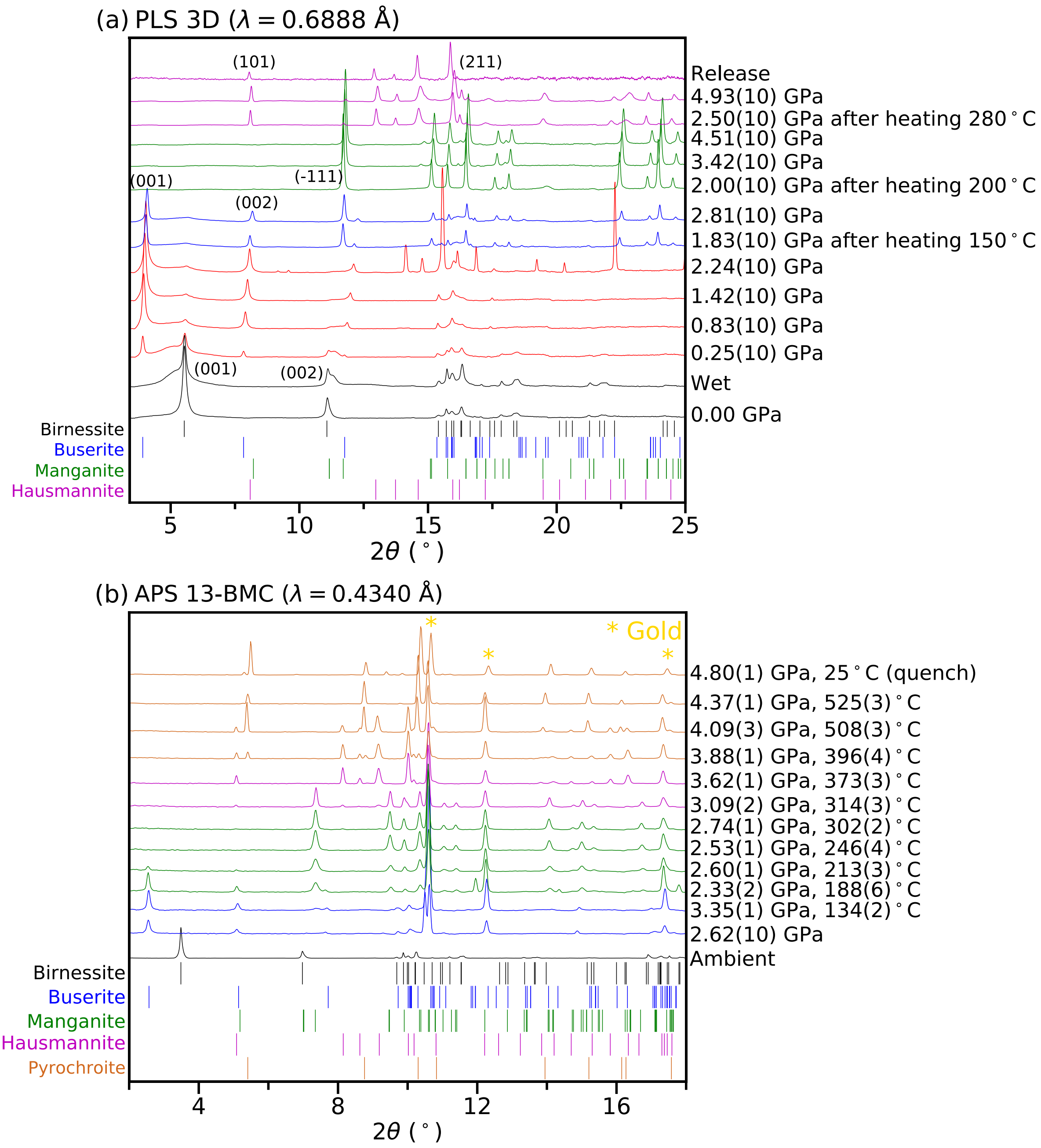

Supplementary Figure 2. Changes in the synchrotron X-ray powder diffraction patterns of birnessite as a function of increasing pressure and temperature under water medium (a) *ex-situ* experiments performed at the 3D beamline at PLS-II and (b) *in-situ* experiments performed at the 13-BMC beamline at APS. All the XRD patterns presented here are after background subtraction for better presentation.

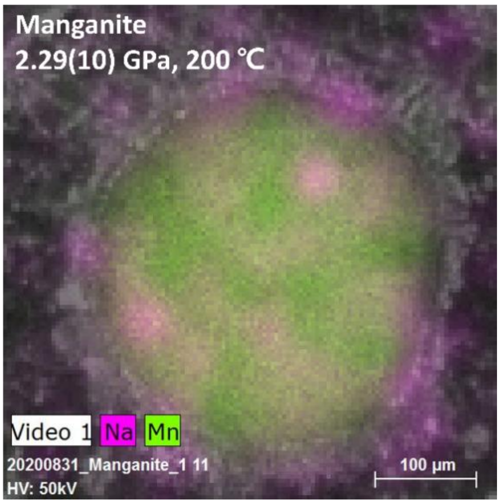

Supplementary Figure 3. Micro-XRF analysis of the recovered manganite from 2.29(10) GPa and 200 ℃. The circular feature outlines the rim of ca. 325 μm diameter gasket used in the DAC experiment.

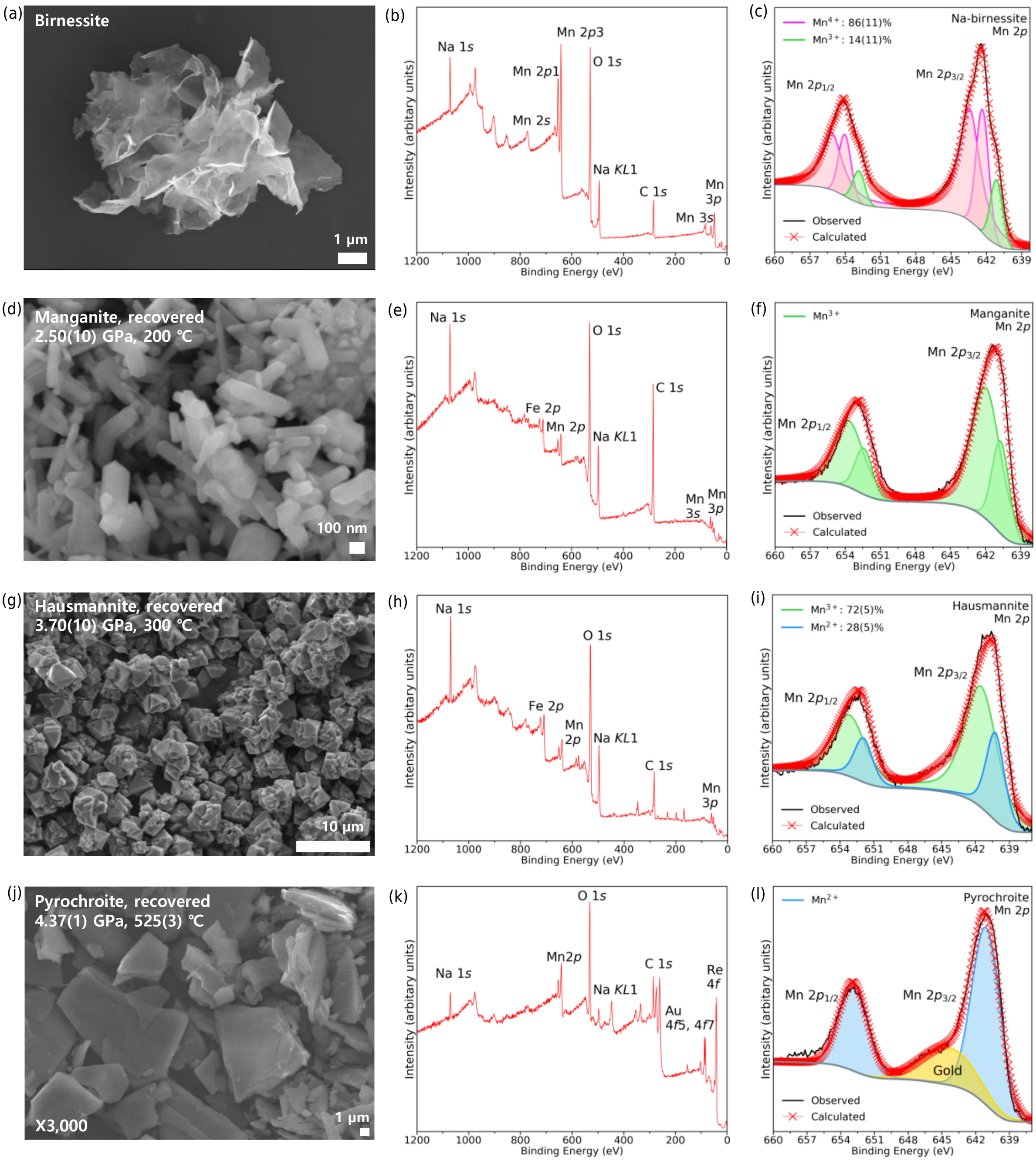

Supplementary Figure 4. SEM images and XPS spectra of (a-c) the original birnessite, (d-f) the recovered manganite, (g-i) hausmannite, and (j-l) pyrochroite. The pressures and temperatures from which each transformation product was recovered are indicated in each data.

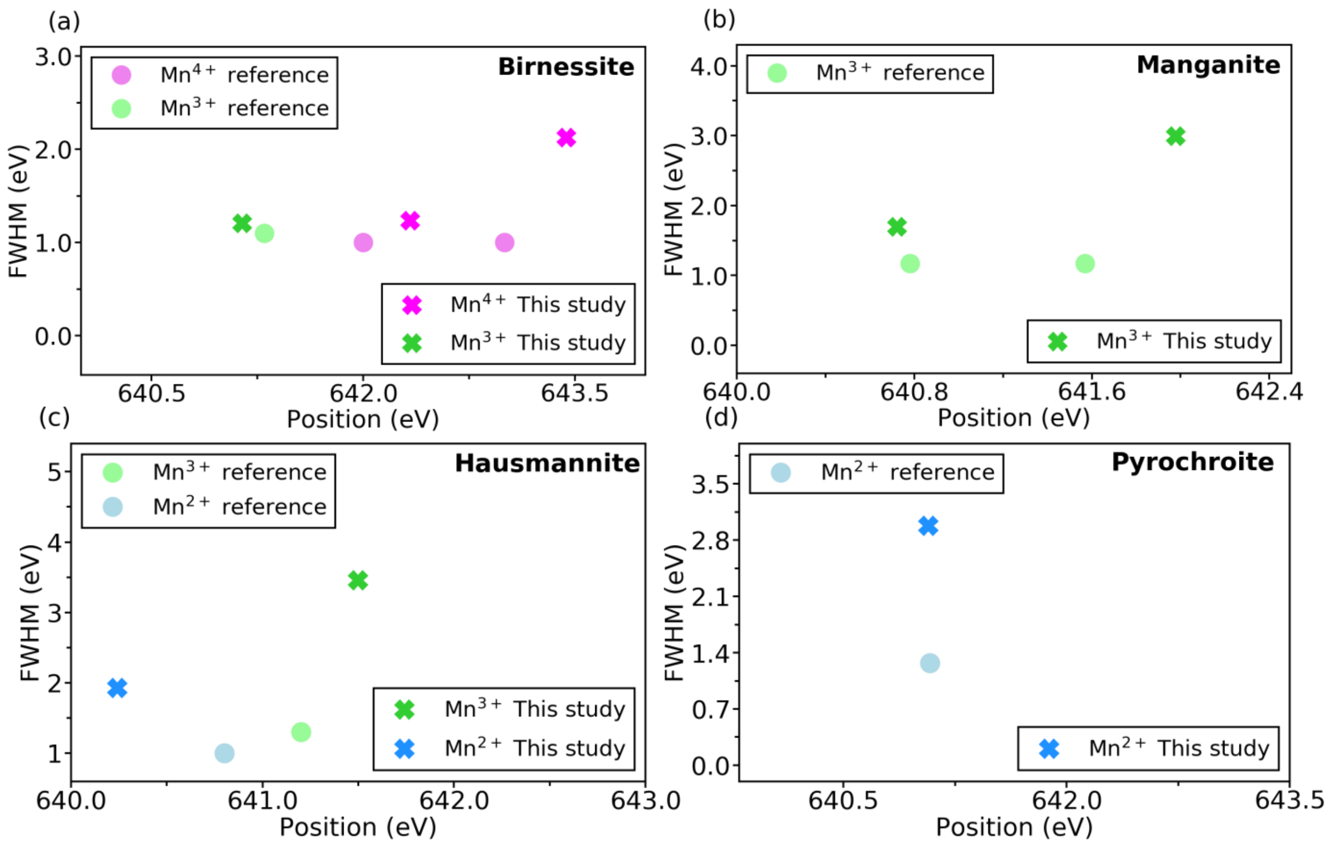

Supplementary Figure 5. The agreement in the Mn^{4+}, Mn^{3+} and Mn^{2+} assignments between the literature[3,4,5,6,7] and our study in terms of Mn2$p_{3/2}$ fitted FWHM (full-width at half-maximum). The original and fitted XPS spectra are shown in Supplementary Fig. 4.

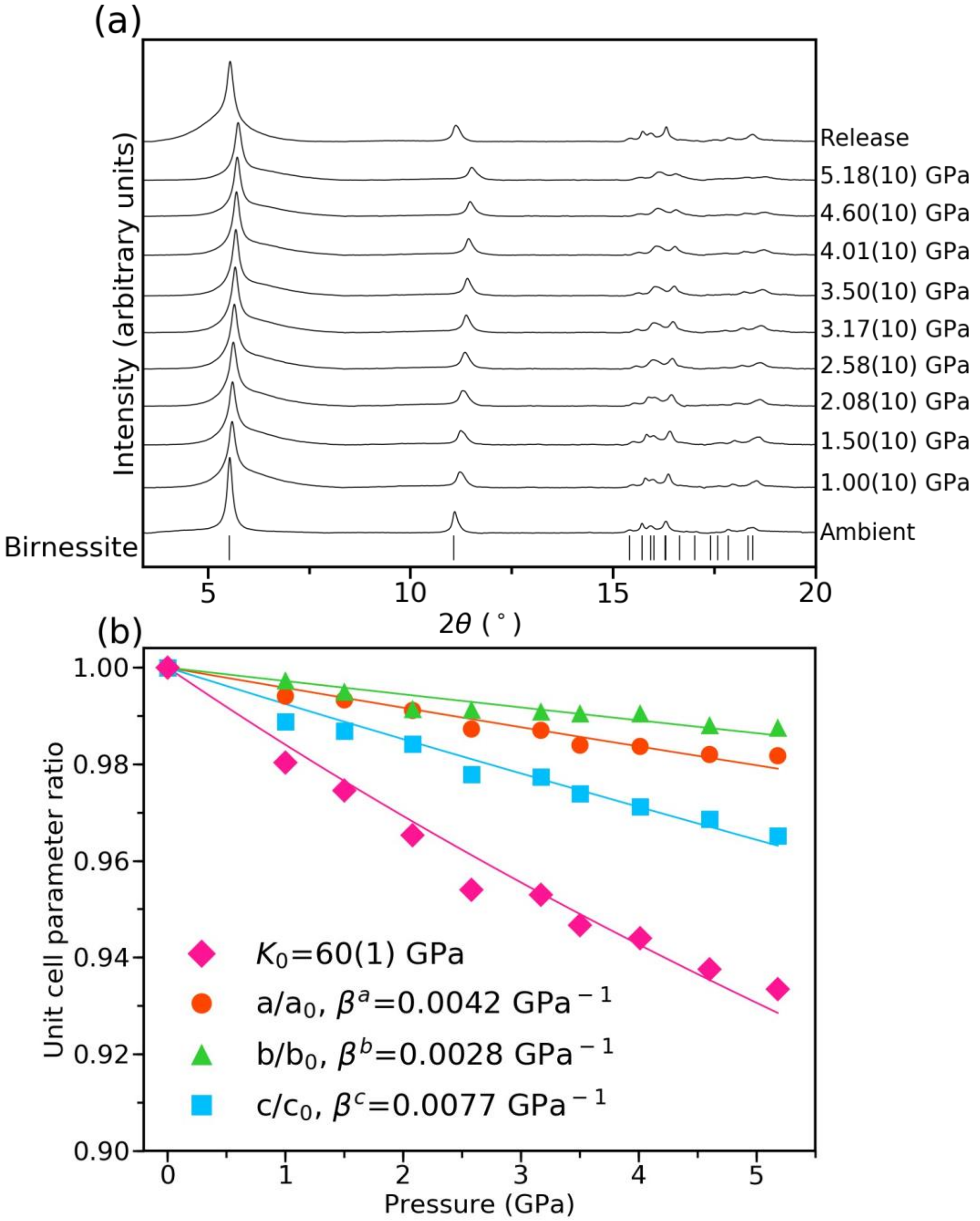

Supplementary Figure 6. (a) Pressure-dependent changes in the XRD patterns of birnessite in silicone oil as a pressure-transmitting medium. (b) Pressure-dependent changes of the normalized unit cell lengths and volume of birnessite in a silicone oil pressure-transmitting medium.

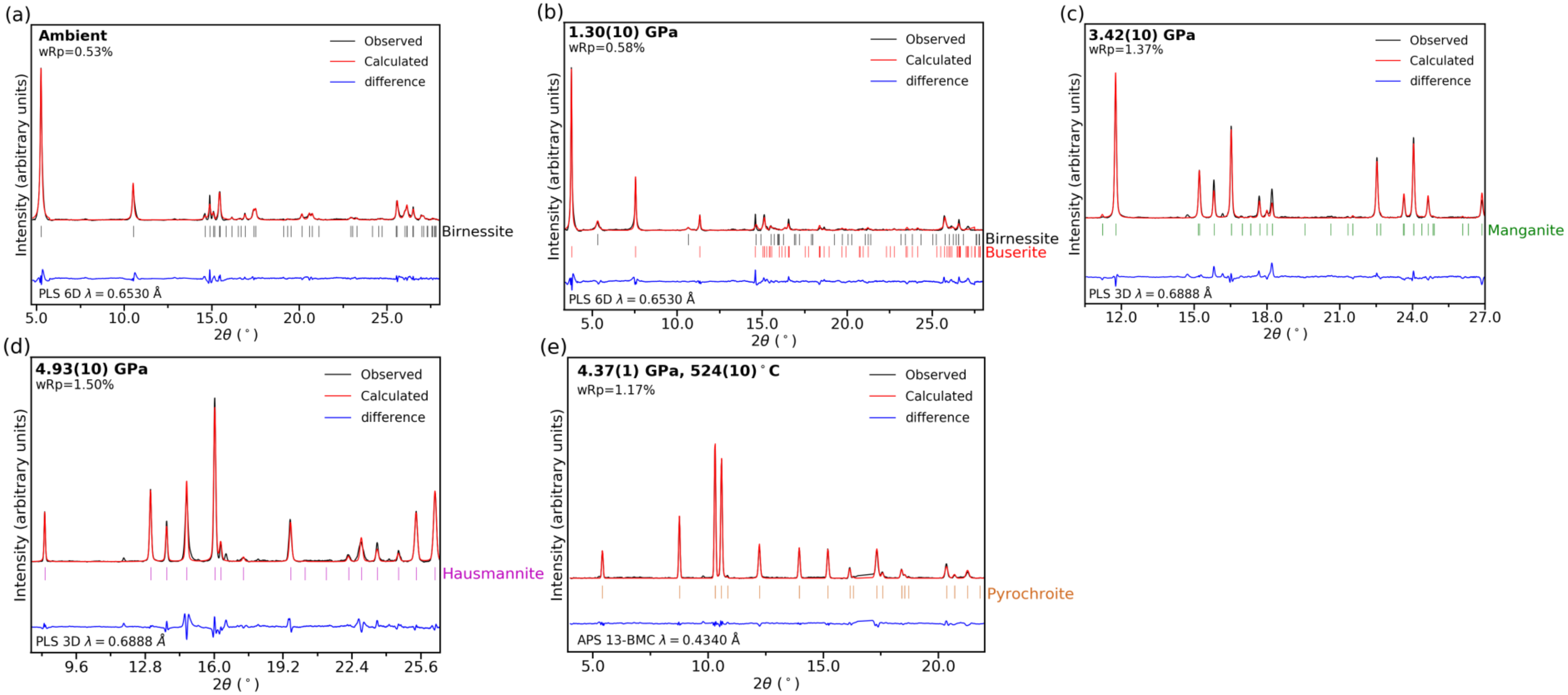

Supplementary Figure 7. Final Rietveld fits for (a) birnessite, (b) buserite, (c) manganite, (d) hausmannite, and (e) pyrochroite. All the XRD patterns presented here are after background subtraction. Pyrochroite data used for the refinement were measured *in-situ*.

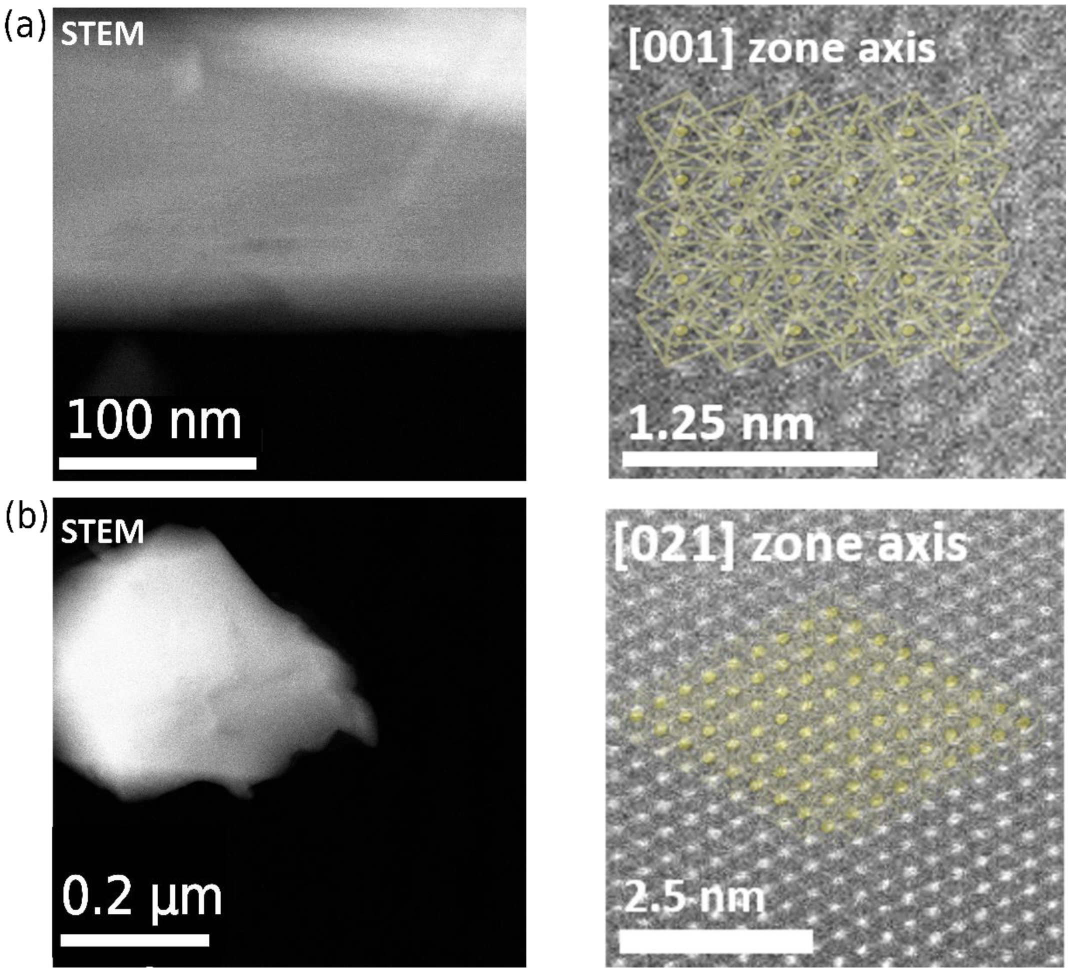

Supplementary Figure 8. (a) STEM images of manganite recovered from 4.20(10) GPa and 200 °C. The structural model of manganite is overlain on the expanded image along the [102] zone axis. (b) STEM images of hausmannite recovered from 6.92(10) GPa and 300 °C. The structural models of manganite and hausmannite are overlain on the expanded STEM images in the right along the [001] and [021] zone axes, respectively.

Supplementary References

1. Rea, D. K. & Ruff, L. J. Composition and mass flux of sediment entering the world's subduction zones: Implications for global sediment budgets, great earthquakes, and volcanism. *Earth Planet. Sci. Lett.* **140**, 1–12 (1996).
2. Plank, T. & Langmuir, C. H. The chemical composition of subducting sediment and its consequences for the crust and mantle. *Chem. Geol.* **145**, 325–394 (1998).
3. Ilton, E. S., Post, J. E., Heaney, P. J., Ling, F. T. & Kerisit, S. N. XPS determination of Mn oxidation states in Mn (hydr) oxides. *Appl. Surf. Sci.* **366**, 475–485 (2016).
4. Boumaiza, H. *et al.* A multi-technique approach for studying Na triclinic and hexagonal birnessites. *J. Solid State Chem.* **272**, 234–243 (2019).
5. Tang, W. *et al.* Oxalate route for promoting activity of manganese oxide catalysts in total VOCs oxidation: effect of calcination temperature and preparation method. *J. Mater. Chem. A* **2**, 2544–2554 (2014).
6. Xing, X. *et al.* Manganese vanadium oxide-N-doped reduced graphene oxide composites as oxygen reduction and oxygen evolution electrocatalysts. *ACS Appl. Mater. Interfaces* **10**, 44511–44517 (2018).
7. Yang, H. Bin. The Structural and Morphology of (La0. 6Sr0. 4) MnO3 Thin Films Prepared by Pulsed Laser Deposition. in *MATEC Web of Conferences* **44**, 2035 (EDP Sciences, 2016).
